# Immune checkpoint inhibitor-induced isolated adrenocorticotropic hormone deficiency: a systematic review

**DOI:** 10.3389/fendo.2024.1326684

**Published:** 2024-01-22

**Authors:** Fen Wang, Xiaoli Shi, Xuefeng Yu, Yan Yang

**Affiliations:** Division of Endocrinology, Tongji Hospital, Tongji Medical College, Huazhong University of Science and Technology, Branch of National Clinical Research Center for Metabolic Diseases, Wuhan, China

**Keywords:** immune checkpoint inhibitor, isolated adrenocorticotropic hormone deficiency, pituitary stimulation test, secondary adrenal insufficiency, provocative test

## Abstract

**Background:**

Immune checkpoint inhibitor-induced isolated adrenocorticotropic hormone deficiency (IAD) is a rare but potentially fatal disease.

**Methods:**

We comprehensively searched the PubMed database and made a systematic review of immune checkpoint inhibitor-induced isolated adrenocorticotropic hormone deficiency. If the status of other anterior pituitary hormones was not mentioned, the case was excluded.

**Results:**

We identified 123 cases diagnosed as immune checkpoint inhibitor-induced IAD, consisting of 44 female and 79 male patients. The average age of these patients was 64.3 ± 12.6 years old, and 67.5% were 60 years old or above. The majority (78.9%) of these patients received anti-programmed cell death protein-1 (anti-PD-1) antibodies or anti-programmed cell death ligand 1 (anti-PD-L1) antibodies or both, and 19.5% received combined therapy, sequential therapy, or both. A total of 26 patients received anti-cytotoxic T lymphocyte antigen 4 antibodies (anti-CTLA-4). The median ICI treatment cycle before the diagnosis of adrenal insufficiency was 8 (6, 12), and the median ICI treatment duration before the diagnosis of adrenal insufficiency was 6 (4, 8) months. Eleven cases developed IAD 1 to 11 months after discontinuation of ICIs. Fatigue and appetite loss were the most common symptoms, and surprisingly, there were two asymptomatic cases of IAD. Most patients (88 cases) had normal pituitary magnetic resonance imaging, only 14 cases reported mild atrophy or swelling pituitary gland, and 21 cases reported no imaging results. Most diagnoses were made by basal hormone levels, and pituitary stimulation tests were performed in only a part of the cases. No cases had been reported of discontinuation of ICI use due to IAD nor had there been any deaths due to IAD.

**Conclusion:**

IAD was predominant in elderly male patients mainly receiving anti-PD-1 or anti-PD-L1 antibodies. It was sometimes difficult to recognize IAD at first glance since non-specific symptoms were common and asymptomatic cases of IAD were also reported. Although IAD can be deadly, it usually does not affect the continued use of ICIs.

## Introduction

Checkpoint inhibitor immunotherapy has been widely used in many kinds of tumors, such as malignant melanoma, lung cancer, advanced urothelial carcinoma, ovarian cancer, and gastric cancer. Immune checkpoint inhibitors (ICIs) are classified into two groups, namely, anti-cytotoxic T-lymphocyte antigen 4 (CTLA-4) antibody represented by ipilimumab and anti-programmed cell death protein-1 (PD-1) antibody and anti-programmed death-ligand-1 (PD-L1) antibody represented by nivolumab and atezolizumab (1). Adverse effects of immune checkpoint inhibitors, particularly immune-related adverse events (irAEs), are increasingly recognized. IrAE involves many organs, for example, the lung, skin, gastrointestinal tract, liver, and endocrine glands. The most common irAE of the endocrine system is hypothyroidism, followed by adrenal insufficiency, hypophysitis, hyperthyroidism, and diabetes (2). Among them, isolated adrenocorticotropin (ACTH) deficiency (IAD) is rare and hard to diagnose promptly. In addition, IAD is usually reported in cases, other than in reviews. We comprehensively searched the PubMed database and made a systematic review of ICI-induced IAD.

## Method

We followed the Preferred Reporting Items for Systematic Reviews and Meta-analyses (PRISMA) reporting guideline.

**Figure 1:**
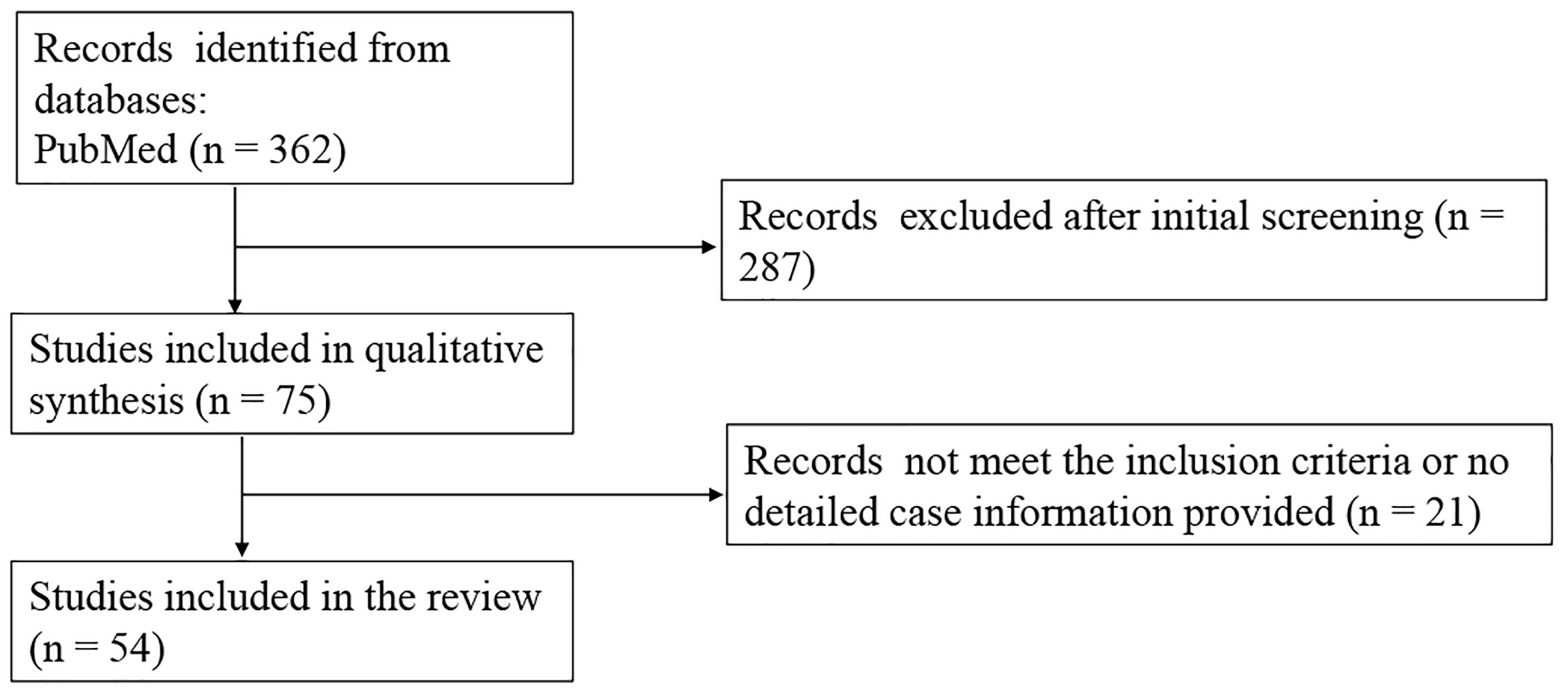
The flowchart for the literature search and review.

Relevant literature was searched through PubMed until 1 May 2023. We also searched the reference lists of the retrieved articles. Keywords were (((((secondary adrenal insufficiency)) OR (isolated ACTH deficiency)) OR (isolated adrenocorticotropic hormone syndrome)) OR (adrenal insufficiency)) AND (((((((((((((((((((((((sintilimab) OR (toripalimab)) OR (tislelizumab)) OR (camrelizumab)) OR (penpulimab)) OR (sugemalimab)) OR (serplulimab)) OR (zimberelimab)) OR (pucotenlimab)) OR (envafolimab)) OR (adebrelimab)) OR (candonilimab)) OR (avelumab)) OR (nivolumab)) OR (pembrolizumab)) OR (ipilimumab)) OR (durvalumab)) OR (atezolizumab)) OR (immune checkpoint inhibitors)) OR (PD-1)) OR (PD-L1)) OR (CTLA4))). The inclusion criteria were as follows: 1) meet the diagnosis of secondary adrenal deficiency; 2) normal function of other anterior pituitary lobes including normal basal growth hormone (GH) and insulin-like growth factor 1 (IGF-1) levels if necessary, thyroid function, and sexual hormone concentration; and 3) IAD occurred after the use of ICIs. If the evaluation of other anterior pituitary hormones was not mentioned or not complete, the case was excluded. Provocative values were not mandatory. The cases (3–7) were excluded because they did not evaluate GH and IGF-1 concentration. The included and excluded papers are shown in **Supplementary Table 1**. The flowchart for the literature search and review is shown in **Figure 1**.

### Statistical analysis

The quantitative data were expressed as the mean ± SD for variables with normal distribution and as the median and interquartile range (IQR) for non-normal variables. The normality of each variable was studied using the Kolmogorov–Smirnov test. Some data in the original paper were represented by line charts, so the specific values were estimated. The value below the lower detection limit was counted as the lower detection limit.

## Results

### Demographic data

A total of 123 cases were diagnosed as immune checkpoint inhibitor-induced IAD, consisting of 44 female and 79 male patients (**Table 1**). The average age of these patients was 64.3 ± 12.6 years old, and 67.5% were 60 years old or above. Most (76.4%) of those cases were reported by Japan, followed by Israel (11.4%), Norway (2.4%), China (1.6%), the United States (1.6%), and Italy (1.6%). Belgium, Spain, Thailand, Turkey, Portugal, and Cameroon reported one case, respectively.

**Table 1:** Basic features of ICIs induced IAD.

	IAD
Number of patients	123
Female: male	44: 79
Ages (years)	64.3 ± 12.6
Tumor type, n (%)	
Melanoma	42 (34.1%)
Lung cancer	38 (30.9%)
Urinary system cancer	23 (18.7%)
Head and neck cancer	8 (6.5%)
Gastric cancer	6 (4.9%)
Breast cancer	2 (1.6%)
Ovary cancer	2 (1.6%)
Cecal cancer	1 (0.8%)
Mesothelioma	1 (0.8%)
ICI, n (%)	
Nivolumab	83 (67.5%)
Pembrolizumab	34 (27.6%)
Ipilimumab	26 (21.1%)
Atezolizumab	3 (2.4%)
Durvalumab	1 (0.8%)
Anti-PD-1 (unknow)	3 (2.4%)
ICI regimen, n (%)	
Monotherapy	99 (80.5%)
Sequential treatment	3 (2.4%)
Combined treatment	15 (12.2%)
Sequential and combined treatment	6 (4.9%)
Time to develop IAD after starting ICI (months), median (IQR)	6 (4, 8)#, N = 95
Number of treatments to develop IAD after starting ICI, median (IQR)	8 (6, 12)*, N = 90

IAD, isolated adrenocorticotropin deficiency; ICIs, Immune checkpoint inhibitors; PD-1, programmed cell death protein-1; #, *: a part of reports did not provide specific time or number of treatments to develop IAD after starting ICI, the data was speculated from the treatment cycle and number of treatments.

### ICIs and IAD

A total of 117 patients (95.1%) received anti-PD-1 antibody treatment, including 83 cases of nivolumab; 34 cases of pembrolizumab; 3 cases of the combination of nivolumab, pembrolizumab, and ipilimumab; and 3 cases of unknown drug. A total of four patients received anti-PD-L1 antibody such as atezolizumab and durvalumab. A total of 26 patients received anti-CTLA-4 antibody treatment, which was ipilimumab. Only two patients received ipilimumab as monotherapy before they developed IAD. Twenty-four patients received sequential treatment, combined treatment, or both treatments. Among them, most patients developed IAD during the treatment with ICIs, and 11 patients developed IAD after stopping ICIs, with a median time of 4.0 (1.0, 6.0) months (**Table 1**).

### Clinical manifestation

The clinical symptoms are diverse. Among them, fatigue occurred in 76.4% cases, followed by appetite loss, nausea, hypotension, and various kinds of pain including myalgia (*n* = 8), arthralgia (*n* = 2), headache (*n* = 2), pericardial chest pain (*n* = 1), back pain (*n* = 1), body pain (*n* = 1), right hypochondrium pain (*n* = 1), and abdominal pain (*n* = 1) (**Table 2**). More than 10% of cases presented hypotension, fever, vomiting, and weight loss. Several patients presented with diarrhea, disturbance of consciousness, somnolence, depression or altered mental status, difficulty walking, malaise, shock, dyspnea, orthostatic hypotension, and dizziness. Abdominal bloating, oliguria, bradykinesia, thirst, dehydration, hiccup, convulsion, asthenia, and vertigo were reported in one case each (**Table 2**).

**Table 2:** The clinical symptoms reported in the IAD cases.

Clinical symptoms	Number (Percent)
Fatigue	94 (76.4%)
Appetite loss	82 (66.7%)
Nausea	24 (19.5%)
Hypotension	18 (14.6%)
Pain	17 (13.8%)
Fever	14 (11.4%)
Vomiting	14 (11.4%)
Weight loss	12 (9.8%)
Diarrhea	9 (7.3%)
Disturbance of consciousness	5 (4.1%)
Somnolence	4 (3.3%)
Depression or altered mental status	4 (3.3%)
Difficulty walking	4 (3.3%)
Malaise	4 (3.3%)
Shock	3 (2.4%)
Dyspnea	2 (1.6%)
Orthostatic hypotension	2 (1.6%)
Dizziness	2 (1.6%)
Abdominal bloating	1 (0.8%)
Oliguria	1 (0.8%)
Bradykinesia	1 (0.8%)
Thirst	1 (0.8%)
Dehydration	1 (0.8%)
Hiccup	1 (0.8%)
Convulsion	1 (0.8%)
Asthenia	1 (0.8%)
Vertigo	1 (0.8%)
No symptom	2 (1.6%)

### Biochemical and imaging results

The ACTH and cortisol concentrations in IAD patients were very low, even below the lower limit of detection. If the value below the detection lower limit was set as the detection lower limit, the median ACTH concentration and cortisol concentration were 5.0 (2.3, 9.3) pg/ml and 1.0 (0.5, 1.6) μg/dl (**Table 3**). Nearly half of the cases (49.6%) reported hyponatremia, 30.1% cases reported eosinophilia, and 10.6% cases reported hypoglycemia. Hyperkalemia was reported in three cases, one due to diabetic ketoacidosis and one due to ileal conduit-related chronic hyperchloremic metabolic acidosis and chronic kidney disease. Hypercalcemia and elevated creatine kinase were also seldom reported. Anti-pituitary cell antibodies were negative in nine cases, and no positive ones were reported. A total of 102 cases reported magnetic resonance imaging (MRI) results of the pituitary gland: 88 cases reported normal pituitary gland, 10 cases reported swelling or enlarged pituitary gland or hypophyseal stalk, and 4 cases reported atrophy of the pituitary gland.

**Table 3:** The biochemical results of IAD.

Item	Number (%)
Hyponatremia	61 (49.6%)
Eosinophilia	37 (30.1%)
Hypoglycemia	13 (10.6%)
Hyperkalemia	3 (2.4%)
Hypercalcemia	1 (0.8%)
Elevated creatine kinase	1 (0.8%)
Serum sodium, mmol/l	130.0 ± 8.0 mmol/l, n = 53
ACTH, pg/ml	5.0 (2.3, 9.3), n = 99
Cortisol, μg/dl	1.0 (0.5, 1.6) , n = 106

ACTH, adrenocorticotropin.

### Provocative tests

Three kinds of stimulation tests were applied to prove secondary adrenal insufficiency (AI) in the literature, namely, corticotrophin-releasing hormone (CRH) stimulation test, ACTH stimulation test, and insulin hypoglycemia test (IHT). CRH stimulation test results were provided in 31 cases, the ACTH stimulation test was done in 8 cases, and IHT was done in 7 cases, while 58 cases reported none of these tests. Cortisol concentration could not be provoked in the CRH test, the IHT test, and most of the ACTH test. However, two cases reported normal cortisol response to prolonged ACTH test.

Other stimulation tests such as thyrotropin-releasing hormone (TRH) test, luteinizing hormone-releasing hormone (LHRH) test, growth hormone releasing peptide 2 (GHRP-2) test, and growth hormone-releasing factor (GFR) test were done to provoke an intact TSH, luteinizing hormone (LH), or GH response. A total of 20 cases applied either the GHRP-2 test or the GRF test or both. Three cases reported a maximum GH concentration of <16 ng/ml, which was lower than the diagnosis criteria in the GHRP-2 test (8). The time to peak values usually occurred at 15 to 60 min. One case had a low IGF-1 level, while there was a normal GH response in the GHRP-2 test. One case had a basal GH concentration of 16.7 ng/ml which needed no further stimulation test. Sixteen cases reported normal IGF-1 levels without the provocative test.

A total of 20 cases reported the TRH test. In total, two cases reported a maximum TSH concentration of below 3.5 mIU/ml because of hyperthyroidism. The time to peak values usually occurred at 30 min except that one case reporting a maximum TSH concentration of 7.14 mIU/ml at 90 min. Another nine cases reported basal TSH concentrations above 3.5 mIU/ml.

The median and maximum LH and follicle-stimulating hormone (FSH) levels were 43.1 (35.5, 51.5) mIU/ml and 43.0 (24.9, 64.0) mIU/ml, respectively, in the LHRH or GnRH tests (**Table 4**). The median concentration of the other pituitary hormones and the time to peak values are also shown in **Table 4**.

**Table 4:** The summary of provocative test results of IAD patients.

	GHmax	LHmax	FSHmax	PRLmax	TSHmax	ACTHmax
Number	20	20	19	20	22	31
Median ± IQR	33.5 (16.0, 44.1)	43.1 (35.5, 51.5)	43.0 (24.9, 64.0)	90.5 (60.0, 117.6)	20.4 (7.0, 36.3)	5.7 (1.4, 12.0)
Median time of max	30 min	60 min	90 min	30 min	30 min	30 min

ACTH, adrenocorticotropin; FSH, follicle-stimulating hormone; GH, growth hormone; LH, luteinizing hormone; PRL, prolactin; TSH, thyroid-stimulating hormone; max, maximum.

### Concurrence of other endocrine adenopathy

A part of these patients developed other types of endocrine adenopathy including 26 cases of primary hypothyroidism and 7 cases of type 1 diabetes mellitus including 2 cases of fulminant type 1 diabetes mellitus and 1 case of diabetes insipidus. Among the 26 cases of ICI-induced hypothyroidism, 8 cases reported transient thyrotoxicosis. Another seven cases reported thyroiditis without a detailed description. No other types of endocrinopathies were reported.

### Treatment of IAD and outcome

The treatments of IAD include medical interventions in the acute and stable phase. During an adrenal crisis, large doses of hydrocortisone are applied immediately once the diagnosis is confirmed. In addition, if necessary, it is needed to balance water and salt metabolism, stabilize blood pressure, and relieve hypoglycemia. Various atypical symptoms such as pain, hiccup, vertigo, and orthostatic hypotension are usually relieved after the use of glucocorticoids. ICIs are usually stopped during an adrenal crisis. During the stable phase, a physiological dose of glucocorticoid should be taken daily. Hydrocortisone is the preferred kind of glucocorticoid. No case was dead because of ICI-induced IAD in the literature.

### Use of ICIs after IAD

A total of 11 cases discontinued the use of ICIs after the occurrence of IAD. The causes were pancreatitis (*N* = 1), patient’s opinion (*N* = 1), ineffectiveness of ICIs, no further therapy of ICIs required (*N* = 4), and discontinued use of ICIs (*N* = 5). Another two cases discontinued the use of ICIs for Guillain–Barre syndrome (*N* = 1) or interstitial pneumonia (*N* = 1) during the continuous use of ICIs after the occurrence of IAD. A total of 28 cases continued to use ICIs after the onset of IAD. Although most of these cases did not report details of immunotherapy after the onset of IAD, none of the authors advocate for the discontinuation of immunotherapy due to the adverse effects of IAD. None of the cases reported stopping immunotherapy after the onset of IAD.

## Discussion

ICIs include anti-CTLA-4 antibodies, anti-PD-1 antibodies, and anti-PD-L1 antibodies. The representative drugs of anti-CTLA-4 antibody are ipilimumab; the representative drugs of anti-PD-1 antibody are nivolumab, penpulimab, sintilimab, camrelizumab, etc.; and the representative drugs of anti-PD-L1 antibody are atezolizumab, durvalumab, avelumab, sugemalimab, and so on (9). All of these three antibodies help T cells kill tumor cells and produce antitumor effects. At present, ICIs are widely used in a variety of malignant tumors such as melanoma, non-small cell lung cancer, and Hodgkin lymphoma, and they are also used in malignant tumors of the digestive tract and urinary system. With the wide application of ICIs, their side effects have also received widespread attention (1). The side effects include itching, rash, and vitiligo on the skin; diarrhea and colitis in the digestive tract; and increased liver and bile enzymes in the liver. In the lungs, it could present as pneumonia. Renal manifestations include creatinine elevation, renal failure, and interstitial nephritis. In the endocrine system, the manifestations are hypothyroidism, hyperthyroidism, hypophysitis, adrenal hypofunction, diabetes, etc. (1). The FDA Side Effect Reporting System collected case data on ICI-related endocrine system side effects that occurred between 2014 and the first quarter of 2019. The number of cases increased year by year. The number of cases reported in the first quarter of 2019 was 6.3 times higher than in the whole of 2014. The most common endocrine system side effect was hypothyroidism, accounting for 14.14% of the total, followed by adrenal insufficiency (11.66%), pituitaritis (10.99%), hyperthyroidism (7.54%), and diabetes (<1%) (2). The occurrence of endocrine system side effects is often related to the immune response, and the corresponding antibodies can be detected, such as thyroid immunoglobulin antibodies, thyroid peroxidase antibodies, anti-adrenal antibodies, and anti-pituitary anterior cell antibodies. IAD is generally associated with the use of anti-PD-1 and PD-L1 antibodies and, less frequently, anti-CTLA-4 antibodies.

Here, we reported 123 cases of ICI-induced IAD, which was the largest review as we know. To be more accurate, we ruled out some cases if the author did not claim to detect an intact anterior pituitary hormone profile or just provided part of the anterior pituitary hormone profile. In our review, the ratio of male patients to female patients was 1.8. The predominance of male patients in ICI-induced IAD was consistent with the review reported by Iglesias (10) and Hinata (11). The average age at diagnosis of IAD was also similar, which was mainly affected by the time of onset of the tumor. Melanoma and lung cancer accounted for 65.0% of the tumors in ICI-induced IAD due in part to the widespread use of ICIs in both cancers. As ICIs are applied to various malignant tumors, more IADs will occur in other types of cancers. Asian researchers reported the most cases of ICI-induced IAD. Japanese researchers also compared the human leukocyte antigen (HLA) signatures between ICI-induced IAD and idiopathic IAD (IIAD) and found no specific HLAs associated with ICI-induced IAD (12), while IIAD had significantly higher frequencies of HLA-DRB1*09:01, HLA-DQA1*03:02, and DQB1*03:03 than healthy controls. The reason why ICI-induced IADs are more common in Asian patients is unknown, which needs further study.

The majority of IADs were associated with the anti-PD-1 antibody, followed by the anti-CTLA-4 antibody and the anti-PD-L1 antibody. Compared with IAD, the anti-CTLA-4 antibody is more likely to cause hypophysitis (13). Among the different classes of ICI treatment such as monotherapy, sequential therapy, combined therapy, and sequential and combined therapy, monotherapy-induced IAD accounted for the majority. Combined therapy had a higher rate of endocrine adverse effects (AEs) than monotherapy. In a systematic review and meta-analysis by Barroso-Sousa et al. (14), patients who received combination therapy had a higher rate of hypothyroidism than the anti-PD-1 antibody, followed by the anti-CTLA-4 antibody. Patients who received combination therapy also had a higher rate of hyperthyroidism than monotherapy. Although the observed incidence of hypophysitis was 3.2% in anti-CTLA-4 antibody, 0.4% in anti-PD-1 antibody, and less than 0.1% in anti-PD-L1 antibody, the incidence was increased to 6.4% in the combination therapy group. It is a reasonable guess that combined therapy will increase the incidence of IAD. However, further evidence is needed.

The median time to onset of different AEs is not consistent. Weber et al. reported that the median time to skin, hepatic, pulmonary, and gastrointestinal-related AEs was between 5.0 and 8.9 weeks (1). The median time to endocrine AEs was 10.4 weeks (1). The endocrine AEs included hypothyroidism, hyperthyroidism, hypophysitis, etc. In our review, the median time to onset of IAD was 6 months, which was the same as Iglesias reported (10) but later than the endocrine AEs reported by Weber et al. (1).

Fatigue, appetite loss, and nausea were the three most common clinical symptoms of IAD. Some rare symptoms had also been reported such as back pain, difficulty walking, malaise, dyspnea, orthostatic hypotension, abdominal bloating, hiccup, convulsion, and bradykinesia. Andrioli et al. also summarized some atypical symptoms such as recurrent syncope, cholestatic jaundice, and pericardial effusion (15). If these manifestations disappear on steroid replacement, they are related to AI condition. Two cases were diagnosed by hormone results without any symptoms. Hyponatremia was frequently reported in IAD although aldosterone secretion was maintained in these patients. However, reduced glomerular filtration rate, increased antidiuretic hormone secretion (15), and appetite loss contributed to the result of hyponatremia. Eosinophilia could occur from a few days to more than a hundred days before IAD (16, 17). Eosinophilia was also reported in other types of adrenal insufficiency and served as a marker of adrenal insufficiency (18). Although sensitivity and specificity were not good enough, we could also make use of blood sodium and eosinophil levels to make an early diagnosis of adrenal insufficiency.

The first step of diagnosing AI is to detect morning serum cortisol concentration. Morning serum cortisol concentration below 3 μg/dl is virtually diagnostic for AI. However, there are other cutoffs of morning serum cortisol concentration reported such as 5.98 μg/dl (19) and 5 μg/dl (20). Other stimulation tests are applied to diagnose AI such as IHT, ACTH test, prolonged ACTH infusion test, and metyrapone test. IHT and metyrapone test may precipitate an acute adrenal crisis, especially in patients with low morning serum cortisol levels. Prolonged ACTH infusion tests are rarely used today because they are inconvenient and less meaningful than ACTH testing. The second step of diagnosing IAD is to detect morning serum ACTH concentration, which is usually low to normal in secondary AI and high in primary AI. The CRH test is used to differentiate hypothalamic AI and ACTH deficiency. Adequate elevated ACTH concentration in IHT and metyrapone test could exclude secondary AI. The third step is to exclude abnormality of pituitary hormones other than ACTH. The diagnosis of hypopituitarism is complicated, such as growth hormone deficiency (GHD), thyroid-stimulating hormone (TSH) deficiency, and hypogonadotropic hypogonadism (HH). The diagnosis of GHD in adults usually needs GH provocative testing or low IGF-1 concentration with three or more other pituitary hormone deficiencies (21). A normal IGF-1 concentration alone does not exclude the diagnosis of GHD because of a considerable overlap in it between individuals with and without GHD (22). IHT is the gold standard to evaluate GHD. IHT is dangerous for untreated AI patients. The GHRP-2 test is a useful and safe alternative to IHT. GHRP-2 is available in Japan and widely used by Japanese researchers. The results of the GHRP-2 test are classified as non-GHD (peak GH > 16 ng/ml), moderate GHD (9 ng/ml < peak GH ≤ 16 ng/ml), and severe GHD (peak GH ≤ 9ng/ml) (8). Since most of the provocative tests were performed in Japan, the above diagnostic criteria apply to these patients in our review. However, three out of 21 cases reported peak GH concentrations of 2.8 ng/ml, 11 ng/ml, and 12.5 ng/ml, respectively, in the GHRP-2 tests (23–25), which were inadequate responses according to the criteria above. However, the authors still claimed the diagnosis of IAD. This discrepancy may be due to a lack of consensus on GHRP-2 testing. The diagnosis of TSH deficiency usually relies on basal TSH and thyroxine values, as most investigators consider the TRH provocative test to be non-specific. However, there were 23 cases who reported the results of the TRH test, most of which had an adequate response to TRH. The dynamic test such as the GnRH test is also not recommended by the researchers. However, it is difficult to detect TSH and gonadotropin deficiency early, and a reliable way needs to be explored.

Although IAD is a rare complication of ICIs, it also can accompany other endocrine adenopathy. According to a single-center analysis by Chen et al. (26), of 28 cases of ICI-induced IAD, 53.5% of the patients had additional endocrinopathy. Among them, primary hypothyroidism accounted for 46.4%, while fulminant type 1 diabetes mellitus accounted for 7.1%. Although we did not observe a high incidence of other endocrine adenopathy as Chen et al., primary hypothyroidism and type 1 diabetes mellitus were also the first and second most common concurrence in our review. It is not surprising that hypothyroidism ranks first since it is the most common endocrine system side effect. However, the relatively high rate of type 1 diabetes mellitus is surprising since it is a kind of rare side effect. However, this observation needs more evidence support.

IAD can be fatal if not diagnosed and treated promptly. There was no case of death reported due to ICI-induced IAD in the literature. However, it is likely that deaths from IAD are not reported. IAD is unlikely to recover and will require lifelong treatment. Patients with IAD are at long-term risk of adrenal crisis, which can shorten life expectancy. To our surprise, Kobayashi et al. reported that patients with ICI-induced pituitary dysfunction in melanoma and non-small cell lung cancer had better overall survival (27). More evidence is needed to support this conclusion.

## Conclusion

IAD caused by ICIs is rare, and dozens of medical records have been documented, most of which are related to anti-PD-1 and anti-PD-L1 antibodies. Fatigue, poor appetite, and nausea associated with isolated ACTH deficiency need to be distinguished from the usual side effects of immune checkpoint inhibitors. IAD can be fatal, and prompt diagnosis and treatment are critical.

## Author contributions

FW: Data curation, Formal analysis, Methodology, Writing – original draft, Writing – review & editing. XS: Writing – review & editing. XY: Writing – review & editing. YY: Investigation, Supervision, Writing – review & editing.
