# Supplementary material for: Immune checkpoint inhibitor-induced isolated adrenocorticotropic hormone deficiency: a systematic review
Source: Front Endocrinol (Lausanne). 2024 Jan 22;15:1326684. doi: 10.3389/fendo.2024.1326684 (PMC10838970; doi:10.3389/fendo.2024.1326684)
Supplement: Supplementary file 1 [file Table_1.docx]

| Supplementary Table 1 Summary of reported cases of ICIs-induced IAD | | | | | | | | |
| --- | --- | --- | --- | --- | --- | --- | --- | --- |
| Author, year | Gender | Age | Tumor | Other endocrine irAEs | ACTH, pg/ml | Cortisol, μg/dl | ICI therapy, cycles | Therapy months |
| Antoniou et al., 2021^[1]^ | Male | 53 | Melanoma |  | 1 | 0.5 | Nivolumab, 21 |  |
| Ariyasu et al., 2017^[2]^ | Male | 60 | Non-small cell lung cancer |  | 6 | 0.6 | Anti-PD-1 | 5 |
| Ariyasu et al., 2017^[2]^ | Male | 72 | Non-small cell lung cancer |  | < 2.0 | 0.3 | Anti-PD-1 | 4 |
| Ariyasu et al., 2017^[2]^ | Male | 71 | Small cell lung cancer | T1DM | 8.5 | 3.2 | Anti-PD-1 | 4 |
| Ariyasu et al., 2018^[3]^ | Female | 63 | Melanoma | Primary hypothyroidism | < 1.0 | 3.5 | Nivolumab, 8 > ipilimumab, 2 | 9 |
| Bekki et al., 2020^[4]^ | Female | 65 | Cecal cancer |  | 3 | 0.5 | Pembrolizumab, 2 |  |
| Boudjemaa et al., 2018 | Male | 60 | Lung carcinoma | Hyperthyroidism | 2.3 | 0.11 | Pembrolizumab, 24 | 39 |
| Cho et al., 2017^[5]^ | Female | 76 | Melanoma |  | 6.7 | < 1 | Nivolumab, 9 | 6 |
| Cho et al., 2017^[5]^ | Male | 54 | Lung carcinoma |  | 7 | < 1 | Nivolumab, 8 | 6 |
| Cho et al., 2017^[5]^ | Male | 64 | Lung adenocarcinoma |  | < 2 | < 1 | Nivolumab, 7 | 4 |
| Cho et al., 2017^[5]^ | Male | 57 | Large cell lung carcinoma |  | 2.9 | < 1 | Nivolumab, 6 | 5 |
| Doodnauth et al., 2021^[6]^ | Male | 85 | Urothelial cancer | Primary hypothyroidism | < 2 | 0.7 | Pembrolizumab, 3 | 3 |
| Fujimura et al., 2017^[7]^ | Male | 68 | Acral lentiginous melanoma |  | < 1.0 | < 0.8 | Nivolumab, 8 | 6 |
| Furubayashi et al., 2018^[8]^ | Female | 74 | Renal clear cell carcinoma |  | 4.5 | 0.1 | Nivolumab, 14 | 7 |
| Hata et al., 2021^[9]^ | Female | 81 | Malignant mesothelioma |  | 3.4 | 1.37 | Nivolumab, 8 | 2 |
| Heck et al., 2021^[10]^ | Female | 64 | Melanoma | Hypothyroidism | 2.27 | 3.82 | Ipilimumab, 4 | 4 |
| Heck et al., 2021^[10]^ | Male | 55 | Melanoma | ICIs-induced hyperthyroidism, followed by hypothyroidism | NA | 1.29 | Ipilimumab and nivolumab | 5 |
| Heck et al., 2021^[10]^ | Female | 40 | Melanoma | Painful thyroid  enlargement and hyperthyroidism |  | 0.29 | Ipilimumab and nivolumab | 2 |
| Hihara et al., 2019^[11]^ | Male | 50 | Head and neck squamous cell cancer |  | 2.8 | 0.2 | Nivolumab, 8 | 4 |
| Hinata et al., 2021^[12]^ | Female | 78 | Advanced ureteral cancer |  | 16.6 | 1.4 | Pembrolizumab, 7 | 5 |
| Inaba et al., 2019^[13]^ | Male | 67 | Lung cancer | DI | 1.7 | <1 | Nivolumab | 7 |
| Inaba et al., 2019^[13]^ | Female | 66 | Melanoma |  | 4.5 | 0.3 | Nivolumab | 5 |
| Inaba et al., 2019^[13]^ | Male | 90 | Lung cancer | ICIs-induced thyroiditis followed by primary hypothyroidism | 8.4 | 1.6 | Pembrolizumab | 5 |
| Inaba et al., 2019^[13]^ | Female | 34 | Parotid cancer |  | 6.5 | 0.1 | Nivolumab | 3 |
| Inaba et al., 2019^[13]^ | Male | 75 | Lung cancer | ICIs-induced thyroiditis | 11 | 1.7 | Pembrolizumab | 3 |
| Inaba et al., 2019^[13]^ | Male | 73 | Melanoma |  | 3.1 | 0.4 | Nivolumab | 6 |
| Inaba et al., 2019^[13]^ | Male | 42 | Gastric cancer |  | 9.1 | 0.6 | Nivolumab | 2 |
| Inaba et al., 2019^[13]^ | Male | 83 | Tongue cancer |  | 10.9 | 1.7 | Nivolumab | 5 |
| Ishikawa et al., 2017^[14]^ | Male | 55 | Melanoma | Hypothyroidism | < 1.0 | 0.5 | Nivolumab, 4 | 3 |
| Kagoshima et al., 2019^[15]^ | Male | 69 | Oropharyngeal squamous cell carcinoma |  | < 2 | 1.38 | Nivolumab, 12 | 6 |
| Kagoshima et al., 2019^[15]^ | Male | 73 | Scc of the epiglottis |  | < 2 | 0.2 | Nivolumab, 10 | 5 |
| Kagoshima et al., 2019^[15]^ | Female | 57 | Scc of the tongue |  | 2.9 | 6.1 | Nivolumab, 9 | 4 |
| Kanie et al., 2017^[16]^ | Male | 65 | Non-small cell lung cancer |  | 3.5 | 0.2 | Atezolizumab, 19 | 14 |
| Kanie et al., 2017^[16]^ | Male | 70 | Non-small cell lung cancer |  | 13.7 | 4.9 | Atezolizumab, 18 | 13 |
| Kitajima et al., 2017^[17]^ | Male | 39 | Melanoma |  | 6.9 | 0.3 | Nivolumab, 13 | 9 |
| Kitajima et al., 2017^[17]^ | Female | 50 | Melanoma |  | < 1.0 | < 1.0 | Nivolumab, 13＞ipilimumab, 2 | 9 |
| Kitano et al., 2018^[18]^ | Male | 53 | Melanoma |  | 6.5 | 0.4 | Nivolumab | 9 |
| Kitano et al., 2018^[18]^ | Male | 72 | Melanoma |  |  |  | Nivolumab | 11 |
| Kurokawa et al., 2022^[19]^ | Male | 70 | Non-small-cell lung cancer | Thyroid dysfunction | 10.1 | 2.4 | Pembrolizumab, 8 | 10 |
| Kurokawa et al., 2022^[19]^ | Female | 49 | Non-small-cell lung cancer | Thyroid dysfunction | ≤ 3 | 4.8 | Pembrolizumab, 14 | 15 |
| Kurokawa et al., 2022^[19]^ | Male | 52 | Non-small-cell lung cancer |  | < 1.5 | 0.6 | Pembrolizumab, 7 | 11 |
| Kurokawa et al., 2022^[19]^ | Female | 80 | Non-small-cell lung cancer |  | 4.2 | 0.6 | Pembrolizumab, 8 | 7 |
| Kurokawa et al., 2022^[19]^ | Female | 67 | Non-small-cell lung cancer |  | 1.5 | 1.4 | Pembrolizumab, 7 | 7 |
| Lupi et al., 2019^[20]^ | Female | 43 | Melanoma | T1DM, transient thyrotoxicosis followed by autoimmune primary hypothyroidism | 5 | 0.8 | Nivolumab > nivolumab and ipilimumab, 1 | 6 |
| Lupi et al., 2019^[20]^ | Male | 80 | Melanoma | Transient thyrotoxicosis followed by autoimmune primary hypothyroidism | < 5 | 0.4 | Pembrolizumab, 9 | 10 |
| Manaka et al., 2021^[21]^ | Male | 55 | Renal cell carcinoma |  | 1.6 | 0.6 | Nivolumab and ipilimumab > nivolumab | 3 |
| Manaka et al., 2021^[21]^ | Male | 76 | Renal cell carcinoma |  | 3.5 | 0.3 | Nivolumab and ipilimumab > nivolumab | 4 |
| Manaka et al., 2021^[21]^ | Male | 73 | Bladder carcinoma |  | 1.2 | 0.3 | Pembrolizumab | 6 |
| Manaka et al., 2021^[21]^ | Male | 74 | Bladder carcinoma |  | 8.1 | 2.2 | Pembrolizumab | 6 |
| Manaka et al., 2021^[21]^ | Male | 42 | Melanoma | Thyroiditis on us | 9 | 2.8 | Pembrolizumab | 16 |
| Martins Machado et al., 2019^[22]^ | Male | 50 | Non-small-cell lung cancer | Thyrotoxicosis followed by primary hypothyroidism | < 0.05 | 0.4 | Nivolumab, 33 | 16 |
| Mishima et al., 2019^[23]^ | Male | 63 | Pulmonary adenocarcinoma | ICIs-induced destructive hyperthyroidism, followed by primary hypothyroidism | < 1.0 | < 0.2 | Nivolumab, 7 | 4 |
| Nagai et al., 2021^[24]^ | Male | 75 | Urothelial carcinoma |  | < 1.5 | 1 | Pembrolizumab, 6 |  |
| Namikawa et al., 2020^[25]^ | Male | 68 | Gastric cancer |  | 3.4 | 0.565 | Nivolumab, 4 | 2 |
| Narahira er al., 2017^[26]^ | Female | 76 | Melanoma |  | 7.18 | < 1.0 | Nivolumab, 9 | 7 |
| Nishio et al., 2021^[27]^ | Female | 34 | Melanoma | Primary hypothyroidism | 5.9 | 0.9 | Nivolumab, 6 | 8 |
| Njonnou et al., 2022^[28]^ | Male | 70 | Melanoma |  | 5.8 | 0.94 | Nivolumab, 8 |  |
| Oguz et al., 2021^[29]^ | Male | 49 | Laryngeal cancer |  | 10.1 | 0.47 | Pembrolizumab, 10 | 9 |
| Ohara et al., 2018^[30]^ | Female | 63 | Lung adenocarcinoma |  | 3.1 | 1.6 | Nivolumab, 17 | 8 |
| Ohara et al., 2019^[31]^ | Female | 69 | Lung adenocarcinoma | Thyrotoxicosis followed by primary hypothyroidism | 2.6 | < 0.2 | Nivolumab, 6 | 6 |
| Okahata et al., 2019^[32]^ | Male | 52 | Breast cancer | FDM | 0.39 | < 1 | Nivolumab, 10 | 6 |
| Okano et al., 2016^[33]^ | Male | 50 | Melanoma |  | 4.9 | 1.7 | Nivolumab, 6 |  |
| Omata et al., 2022^[34]^ | Male | 83 | Melanoma |  | 12.6 | 1.26 | Nivolumab, 17 | 8 |
| Ono et al., 2022^[35]^ | Male | 63 | Non-small cell lung cancer |  |  |  | Pembrolizumab, 39 |  |
| Ono et al., 2022^[35]^ | Male | 70 | Non-small cell lung cancer | Primary hypothyroidism |  |  | Pembrolizumab, 2 |  |
| Ono et al., 2022^[35]^ | Female | 71 | Non-small cell lung cancer | Primary hypothyroidism |  |  | Atezolizumab, 59 |  |
| Ono et al., 2022^[35]^ | Male | 77 | Non-small cell lung cancer |  |  |  | Pembrolizumab, 14 |  |
| Ono et al., 2022^[35]^ | Male | 78 | Non-small cell lung cancer |  |  |  | Pembrolizumab, 10 |  |
| Ono et al., 2022^[35]^ | Male | 56 | Kidney cancer |  |  |  | Nivolumab, 13 |  |
| Ono et al., 2022^[35]^ | Male | 57 | Kidney cancer |  |  |  | Nivolumab, 4 and ipilimumab, 4 |  |
| Ono et al., 2022^[35]^ | Male | 71 | Kidney cancer |  |  |  | Nivolumab, 5 and ipilimumab, 4 |  |
| Ono et al., 2022^[35]^ | Male | 85 | Kidney cancer |  |  |  | Nivolumab, 8 and ipilimumab, 4 |  |
| Ono et al., 2022^[35]^ | Female | 72 | Melanoma |  |  |  | Nivolumab, 3 and ipilimumab, 3 |  |
| Ono et al., 2022^[35]^ | Male | 83 | Melanoma | T1DM |  |  | Nivolumab, 5 and ipilimumab, 4 |  |
| Ono et al., 2022^[35]^ | Male | 78 | Bladder carcinoma |  |  |  | Pembrolizumab, 10 |  |
| Ono et al., 2022^[35]^ | Male | 66 | Nasal cancer |  |  |  | Pembrolizumab, 9 |  |
| Oristrell et al., 2018^[36]^ | Female | 55 | Breast cancer | Primary hypothyroidism | < 1.6 | 0.93 | Pembrolizumab, 5 | 12 |
| Percik et al., 2020^[37]^ | Male | 65 | Melanoma |  | 11.8 | 4.28 | Pembrolizumab | 16 |
| Percik et al., 2020^[37]^ | Male | 30 | Melanoma |  | 11.9 | 1.13 | Nivolumab and ipilimumab | 3 |
| Percik et al., 2020^[37]^ | Female | 57 | Ovary cancer |  | < 5 | < 1.0 | Durvalumab | 7 |
| Percik et al., 2020^[37]^ | Female | 66 | Melanoma | Thyroiditis | 6 | 1.33 | Nivolumab and ipilimumab | 9 |
| Percik et al., 2020^[37]^ | Female | 68 | Melanoma | Thyroiditis | 5.6 | 2.28 | Pembrolizumab > nivolumab > nivolumab and ipilimumab | 5 |
| Percik et al., 2020^[37]^ | Female | 51 | Breast cancer |  | < 5 | < 1.0 | Pembrolizumab | 6 |
| Percik et al., 2020^[37]^ | Female | 52 | Renal cancer |  | < 5 | < 1.0 | Nivolumab and ipilimumab | 3 |
| Percik et al., 2020^[37]^ | Male | 78 | Melanoma |  | < 5 | < 1.0 | Pembrolizumab > nivolumab and ipilimumab | 6 |
| Percik et al., 2020^[37]^ | Female | 42 | Gastric cancer |  | 7.7 | < 1.0 | Nivolumab and ipilimumab | 3 |
| Percik et al., 2020^[37]^ | Male | 71 | Transitional-cell-carcinoma |  | 13.1 | 1.16 | Pembrolizumab | 6 |
| Percik et al., 2020^[37]^ | Female | 38 | Melanoma | Thyroiditis | < 5 | < 1.0 | Nivolumab and ipilimumab > pembrolizumab | 13 |
| Percik et al., 2020^[37]^ | Female | 59 | Melanoma |  | NA | 1.41 | Ipilimumab | 4 |
| Percik et al., 2020^[37]^ | Female | 58 | Ovary cancer |  | 10.8 | 3.27 | Pembrolizumab | 4 |
| Percik et al., 2020^[37]^ | Female | 60 | Melanoma |  | NA | < 1.0 | Nivolumab and ipilimumab | 4 |
| Pierrard et al., 2019^[38]^ | Male | 70 | Advanced urothelial carcinoma |  | 10 | 1.4 | Nivolumab, 9 | 4 |
| Porntharukchareon et al., 2020^[39]^ | Male | 70 | Non-small cell lung cancer | FDM | 21.7 | 0.8 | Pembrolizumab, 5 and ipilimumab, 3 | 4 |
| Sakaguchi et al., 2019^[40]^ | Female | 60 | Melanoma |  | < 1.0 | < 1.0 | Nivolumab, 13 > ipilimumab, 2 |  |
| Sato et al., 2019^[41]^ | Male | 79 | Squamous cell lung cancer |  | < 1.0 | 1.1 | Nivolumab, 20 | 8 |
| Seki et al., 2017^[42]^ | Female | 74 | Renal clear cell carcinoma | Primary hypothyroidism | 14.4 | 2.34 | Nivolumab, 5 | 3 |
| Shrotriya et al.， 2018^[43]^ | Male | 73 | Egfr positive lung adenocarcinoma |  | < 5 | 0.4 | Nivolumab, 4 |  |
| Suzuki et al., 2020^[44]^ | Male | 68 | Renal clear cell carcinoma | Primary hypothyroidism | 16.3 | 1.9 | Nivolumab, 8 | 3 |
| Suzuki et al., 2020^[44]^ | Male | 87 | Renal clear cell carcinoma | Primary hypothyroidism | 4.2 | 1.9 | Nivolumab, 4 | 2 |
| Suzuki et al., 2020^[44]^ | Female | 69 | Renal clear cell carcinoma |  | 12.2 | 6 | Nivolumab, 6 | 5 |
| Suzuki et al., 2020^[44]^ | Male | 64 | Renal clear cell carcinoma |  | 9.3 | 2.9 | Nivolumab, 10 | 6 |
| Suzuki et al., 2020^[44]^ | Male | 71 | Renal clear cell carcinoma |  | 4.9 | 3.1 | Nivolumab, 3 | 1 |
| Takaya et al., 2017^[45]^ | Male | 75 | Lung adenocarcinoma |  | < 1.0 | 0.4 | Nivolumab, 12 | 6 |
| Takebayashi et al., 2018^[46]^ | Male | 58 | Melanoma | ICIs-induced destructive hyperthyroidism, followed by primary hypothyroidism | < 2 | 0.34 | Nivolumab | 7 |
| Takeno et al.,2019^[47]^ | Female | 72 | Melanoma | Primary hypothyroidism | 9.6 | < 1 | Nivolumab, 14 | 15 |
| Tanabe et al., 2022^[48]^ | Female | 58 | Squamous cell lung cancer |  | 3.2 | 0.8 | Pembrolizumab, 4 |  |
| Tanaka et al., 2020^[49]^ | Male | 85 | Squamous cell lung carcinoma |  | 8.3 | 0.92 | Pembrolizumab, 8 | 6 |
| Tanaka et al., 2020^[49]^ | Female | 85 | Squamous cell lung carcinoma |  | 8.3 | 0.92 | Pembrolizumab, 8 | 6 |
| Yamagata et al., 2019^[50]^ | Male | 59 | Non-small cell lung cancer | Primary hypothyroidism | 17.3 | 0.89 | Pembrolizumab, 5 | 7.5 |
| Yamauchi et al., 2021^[51]^ | Female | 75 | Lung caner |  | 1.4 | 0.22 | Nivolumab, 7 | 9 |
| Yamauchi et al., 2021^[51]^ | Male | 63 | Lung caner |  | 12.7 | 1.58 | Nivolumab, 4 | 6 |
| Yamauchi et al., 2021^[51]^ | Male | 72 | Melanoma | Thyrotoxicosis | 14.1 | 1.87 | Nivolumab, 4 | 2 |
| Yamauchi et al., 2021^[51]^ | Male | 84 | Melanoma | T1DM | 27.7 | 2.88 | Nivolumab, 4 | 2 |
| Yamauchi et al., 2021^[51]^ | Male | 59 | Melanoma | T1DM, thyrotoxicosis followed by primary hypothyroidism | 17.7 | 1.4 | Nivolumab, 7 | 3 |
| Yamauchi et al., 2021^[51]^ | Male | 73 | Gastric cancer |  | 2.7 | 0.68 | Nivolumab, 4 | 5 |
| Yamauchi et al., 2021^[51]^ | Male | 56 | Gastric cancer |  | 2.7 | 0.68 | Nivolumab, 4 | 2 |
| Yano et al., 2020^[52]^ | Female | 67 | Melanoma |  | < 2.0 | 0.5 | Nivolumab, 14 > ipilimumab, 4 |  |
| Yano et al., 2020^[52]^ | Female | 68 | Non-small cell lung cancer |  | 3.1 | 0.9 | Nivolumab, 2 |  |
| Yano et al., 2020^[52]^ | Female | 68 | Melanoma | Primary hypothyroidism | 21 | 3.1 | Ipilimumab, 3 > nivolumab, 7 |  |
| Yano et al., 2020^[52]^ | Male | 58 | Gastric cancer |  | < 2.0 | 0.5 | Nivolumab, 10 |  |
| Yano et al., 2020^[52]^ | Male | 63 | Non-small cell lung cancer |  | < 2.0 | 0.3 | Nivolumab, 11 |  |
| Yano et al., 2020^[52]^ | Female | 50 | Melanoma | Primary hypothyroidism | < 2.0 | < 0.1 | Nivolumab, 13 |  |
| Yano et al., 2020^[52]^ | Male | 39 | Melanoma |  | 9.1 | 0.3 | Nivolumab, 13 |  |
| Zeng et al., 2017^[53]^ | Male | 54 | Renal clear cell carcinoma | Primary hypothyroidism | < 10 | 0.81 | Nivolumab, 12 | 6 |
| Zhu et al., 2019^[54]^ | Male | 61 | Small cell lung cancer | Primary hypothyroidism, | 2.43 | 0.64 | Nivolumab, 6 | 4 |
| DI, diabetes insipidus; FDM, fulminant type 1 diabetes mellitus; ICI, immune checkpoint inhibitor; irAEs, immune related adverse effects; T1DM, type 1 diabetes mellitus; | | | | | | | | |

The reports that we excluded in the second time were shown as the reference 55 to 75 ^[55-75]^. These papers were excluded for not meeting the inclusion criteria or no detailed cases provided.

Reference

1. Antoniou, S., G. Bazazo, L. Röckl, M. Papadakis, and C. Berg, Late-onset hypophysitis after discontinuation of nivolumab treatment for advanced skin melanoma: a case report*.* BMC Endocrine Disorders, 2021. 21(1). <https://doi.org/10.1186/s12902-021-00854-y>.

2. Ariyasu, R., A. Horiike, T. Yoshizawa, Y. Dotsu, J. Koyama, M. Saiki, T. Sonoda, S. Nishikawa, S. Kitazono, N. Yanagitani, and M. Nishio, Adrenal Insufficiency Related to Anti-Programmed Death-1 Therapy*.* Anticancer Res, 2017. 37(8): p. 4229-4232. <https://doi.org/10.21873/anticanres.11814>.

3. Ariyasu, H., H. Inaba, T. Ota, H. Yamaoka, Y. Furukawa, H. Iwakura, N. Doi, Y. Yamamoto, and T. Akamizu, Thyrotoxicosis and Adrenocortical Hormone Deficiency During Immune-checkpoint Inhibitor Treatment for Malignant Melanoma*.* In Vivo, 2018. 32(2): p. 345-351. <https://doi.org/10.21873/invivo.11244>.

4. Bekki, T., Y. Takakura, M. Kochi, Y. Konemori, K. Oki, M. Yoneda, H. Egi, and H. Ohdan, A Case of Isolated Adrenocorticotropic Hormone Deficiency Caused by Pembrolizumab*.* Case Rep Oncol, 2020. 13(1): p. 200-206. <https://doi.org/10.1159/000505687>.

5. Cho, K.Y., H. Miyoshi, A. Nakamura, T. Kurita, and T. Atsumi, Hyponatremia can be a powerful predictor of the development of isolated ACTH deficiency associated with nivolumab treatment [Letter to the Editor]*.* Endocr J, 2017. 64(2): p. 235-236. <https://doi.org/10.1507/endocrj.EJ16-0596>.

6. Doodnauth, A.V., M. Klar, Y.S. Mulatu, Z.R. Malik, K.H. Patel, and S.I. McFarlane, Pembrolizumab-Induced Hypophysitis With Isolated Adrenocorticotropic Hormone (ACTH) Deficiency: A Rare Immune-Mediated Adverse Event*.* Cureus, 2021. 13(6): p. e15465. <https://doi.org/10.7759/cureus.15465>.

7. Fujimura, T., Y. Kambayashi, S. Furudate, A. Kakizaki, T. Hidaka, T. Haga, A. Hashimoto, R. Morimoto, and S. Aiba, Isolated adrenocorticotropic hormone deficiency possibly caused by nivolumab in a metastatic melanoma patient*.* J Dermatol, 2017. 44(3): p. e13-e14. <https://doi.org/10.1111/1346-8138.13532>.

8. Furubayashi, N., T. Negishi, T. Uozumi, D. Takamatsu, K. Shiraishi, D. Hirose, and M. Nakamura, Isolated adrenocorticotropic hormone deficiency potentially induced by nivolumab following pseudo-progression in clear cell renal cell carcinoma: A case report*.* Mol Clin Oncol, 2019. 10(2): p. 304-308. <https://doi.org/10.3892/mco.2018.1781>.

9. Hata, K., C. Sakaguchi, M. Tsuchiya, and Y. Nagasaka, Abdominal pain as an initial symptom of isolated ACTH deficiency induced by nivolumab in a patient with malignant mesothelioma*.* BMJ Case Rep, 2021. 14(7). <https://doi.org/10.1136/bcr-2021-243093>.

10. Heck, A. and A.K. Winge-Main, Silent, isolated ACTH deficiency in malignant melanoma patients treated with immune checkpoint inhibitors*.* BMJ Case Rep, 2021. 14(5). <https://doi.org/10.1136/bcr-2021-241981>.

11. Hihara, K., H. Sato, I. Okamoto, Y. Katsube, R. Maruyama, R. Tomioka, H. Tanaka, and K. Tsukahara, Pituitary-adrenal dysfunction caused by nivolumab for head and neck cancer*.* Auris Nasus Larynx, 2019. 46(6): p. 896-901. <https://doi.org/10.1016/j.anl.2019.02.005>.

12. Hinata, Y., N. Ohara, Y. Sakurai, R. Koda, Y. Yoneoka, T. Takada, N. Hara, and T. Nishiyama, Isolated Adrenocorticotropic Hormone Deficiency Associated with Severe Hyperkalemia During Pembrolizumab Therapy in a Patient with Ureteral Cancer and an Ileal Conduit: A Case Report and Literature Review*.* Am J Case Rep, 2021. 22: p. e931639. <https://doi.org/10.12659/AJCR.931639>.

13. Inaba, H., H. Ariyasu, H. Iwakura, Y. Ueda, C. Kurimoto, S. Uraki, K. Takeshima, H. Yamaoka, Y. Furukawa, S. Morita, M. Nishi, and T. Akamizu, Comparative analysis of human leucocyte antigen between idiopathic and anti-PD-1 antibody induced isolated adrenocorticotropic hormone deficiency: A pilot study*.* Clin Endocrinol (Oxf), 2019. 91(6): p. 786-792. <https://doi.org/10.1111/cen.14082>.

14. Ishikawa, M. and K. Oashi, Case of hypophysitis caused by nivolumab*.* J Dermatol, 2017. 44(1): p. 109-110. <https://doi.org/10.1111/1346-8138.13437>.

15. Kagoshima, H., R. Hori, T. Kojima, Y. Okanoue, S. Fujimura, A. Taguchi, and K. Shoji, Adrenal insufficiency following nivolumab therapy in patients with recurrent or metastatic head and neck cancer*.* Auris Nasus Larynx, 2020. 47(2): p. 309-313. <https://doi.org/10.1016/j.anl.2019.05.009>.

16. Kanie, K., G. Iguchi, H. Bando, Y. Fujita, Y. Odake, K. Yoshida, R. Matsumoto, H. Fukuoka, W. Ogawa, and Y. Takahashi, Two Cases of Atezolizumab-Induced Hypophysitis*.* J Endocr Soc, 2018. 2(1): p. 91-95. <https://doi.org/10.1210/js.2017-00414>.

17. Kitajima, K., K. Ashida, N. Wada, R. Suetsugu, Y. Takeichi, S. Sakamoto, H. Uchi, T. Matsushima, M. Shiratsuchi, K. Ohnaka, M. Furue, and M. Nomura, Isolated ACTH deficiency probably induced by autoimmune-related mechanism evoked with nivolumab*.* Jpn J Clin Oncol, 2017. 47(5): p. 463-466. <https://doi.org/10.1093/jjco/hyx018>.

18. Kitano, S., K. Tatsuno, J. Ishibe, T. Shimauchi, T. Fujiyama, T. Ito, N. Ogawa, and Y. Tokura, Isolated Adrenocorticotropic Hormone Deficiency in Melanoma Patients Treated with Nivolumab*.* Acta Derm Venereol, 2018. 98(7): p. 704-705. <https://doi.org/10.2340/00015555-2902>.

19. Kurokawa, K., Y. Mitsuishi, N. Shimada, N. Ito, M. Ogiwara, K. Miura, T. Asao, R. Ko, T. Shukuya, R. Shibayama, H. Goto, and K. Takahashi, Clinical characteristics of adrenal insufficiency induced by pembrolizumab in non-small-cell lung cancer*.* Thorac Cancer, 2023. 14(5): p. 442-449. <https://doi.org/10.1111/1759-7714.14761>.

20. Lupi, I., A. Brancatella, M. Cosottini, N. Viola, G. Lanzolla, D. Sgro, G.D. Dalmazi, F. Latrofa, P. Caturegli, and C. Marcocci, Clinical heterogeneity of hypophysitis secondary to PD-1/PD-L1 blockade: insights from four cases*.* Endocrinol Diabetes Metab Case Rep, 2019. 2019. <https://doi.org/10.1530/EDM-19-0102>.

21. Manaka, K., J. Sato, M. Takeuchi, K. Watanabe, H. Kage, T. Kawai, Y. Sato, T. Miyagawa, D. Yamada, H. Kume, S. Sato, T. Nagase, T. Iiri, M. Nangaku, and N. Makita, Immune checkpoint inhibitor combination therapies very frequently induce secondary adrenal insufficiency*.* Sci Rep, 2021. 11(1): p. 11617. <https://doi.org/10.1038/s41598-021-91032-6>.

22. Martins Machado, C., L. Almeida Santos, A. Barroso, and M.J. Oliveira, Nivolumab-induced hypothyroidism followed by isolated ACTH deficiency*.* BMJ Case Rep, 2019. 12(8). <https://doi.org/10.1136/bcr-2019-231236>.

23. Mishima, Y., T. Fukaishi, N. Inase, and S. Isogai, Nivolumab-induced Hypophysitis, Secondary Adrenal Insufficiency and Destructive Thyroiditis in a Patient with Lung Adenocarcinoma*.* Intern Med, 2019. 58(5): p. 693-697. <https://doi.org/10.2169/internalmedicine.1268-18>.

24. Nagai, T., T. Mogami, T. Takeda, N. Tomiyama, and T. Yasui, A case of secondary adrenocortical insufficiency due to isolated adrenocorticotropic hormone deficiency with empty sella syndrome after pembrolizumab treatment in a patient with metastatic renal pelvic cancer*.* Urol Case Rep, 2021. 39: p. 101766. <https://doi.org/10.1016/j.eucr.2021.101766>.

25. Namikawa, T., S. Shimizu, K. Yokota, N. Tanioka, I. Fukudome, M. Munekage, S. Uemura, H. Maeda, H. Kitagawa, and K. Hanazaki, Isolated adrenocorticotropic hormone deficiency induced by nivolumab treatment for advanced gastric cancer*.* Clin J Gastroenterol, 2021. 14(4): p. 988-993. <https://doi.org/10.1007/s12328-021-01384-9>.

26. Narahira, A., T. Yanagi, K.Y. Cho, A. Nakamura, H. Miyoshi, H. Hata, K. Imafuku, S. Kitamura, and H. Shimizu, Isolated adrenocorticotropic hormone deficiency associated with nivolumab therapy*.* J Dermatol, 2017. 44(4): p. e70. <https://doi.org/10.1111/1346-8138.13571>.

27. Nishio, K., Y. Okada, A. Kurozumi, and Y. Tanaka, A Case of Thyroid Dysfunction and Isolated Adrenocorticotropin Deficiency after Nivolumab Therapy for Malignant Melanoma*.* J UOEH, 2021. 43(1): p. 97-102. <https://doi.org/10.7888/juoeh.43.97>.

28. Simeni Njonnou, S.R., S. Aspeslagh, M.J. Ntsama Essomba, M.L. Racu, F. Kemta Lekpa, and F. Vandergheynst, Isolated adrenocorticotropic hormone deficiency and sialadenitis associated with nivolumab: a case report*.* J Med Case Rep, 2022. 16(1): p. 456. <https://doi.org/10.1186/s13256-022-03663-6>.

29. Oguz, S.H., U. Unluturk, S. Aksoy, and T. Erbas, Clinical course and management of pembrolizumab-associated isolated adrenocorticotrophic hormone deficiency: a new case and literature review*.* Immunotherapy, 2021. 13(14): p. 1157-1163. <https://doi.org/10.2217/imt-2021-0061>.

30. Ohara, N., K. Ohashi, T. Fujisaki, C. Oda, Y. Ikeda, Y. Yoneoka, T. Hashimoto, G. Hasegawa, K. Suzuki, and T. Takada, Isolated Adrenocorticotropin Deficiency due to Nivolumab-induced Hypophysitis in a Patient with Advanced Lung Adenocarcinoma: A Case Report and Literature Review*.* Intern Med, 2018. 57(4): p. 527-535. <https://doi.org/10.2169/internalmedicine.9074-17>.

31. Ohara, N., M. Kobayashi, K. Ohashi, R. Ito, Y. Ikeda, G. Kawaguchi, Y. Yoneoka, G. Hasegawa, and T. Takada, Isolated adrenocorticotropic hormone deficiency and thyroiditis associated with nivolumab therapy in a patient with advanced lung adenocarcinoma: a case report and review of the literature*.* J Med Case Rep, 2019. 13(1): p. 88. <https://doi.org/10.1186/s13256-019-2002-2>.

32. Okahata, S., K. Sakamoto, T. Mitsumatsu, Y. Kondo, S. Noso, H. Ikegami, and T. Shiba, Fulminant type 1 diabetes associated with Isolated ACTH deficiency induced by anti-programmed cell death 1 antibody-insight into the pathogenesis of autoimmune endocrinopathy*.* Endocr J, 2019. 66(4): p. 295-300. <https://doi.org/10.1507/endocrj.EJ18-0328>.

33. Okano, Y., T. Satoh, K. Horiguchi, M. Toyoda, A. Osaki, S. Matsumoto, T. Tomaru, Y. Nakajima, S. Ishii, A. Ozawa, N. Shibusawa, T. Shimada, T. Higuchi, K. Chikamatsu, and M. Yamada, Nivolumab-induced hypophysitis in a patient with advanced malignant melanoma*.* Endocr J, 2016. 63(10): p. 905-912. <https://doi.org/10.1507/endocrj.EJ16-0161>.

34. Omata, W., S. Nakamura, C. Urasaki, H. Morita, H. Funaishi, K. Kobayashi, H. Koide, A. Tsutsumida, and H. Matsue, Secondary Adrenal Insufficiency in a Patient with Metastatic Melanoma Treated with Nivolumab*.* Case Rep Dermatol, 2022. 14(1): p. 55-60. <https://doi.org/10.1159/000523798>.

35. Ono, M., I. Fukuda, M. Nagao, K. Tomiyama, M. Okazaki-Hada, Y. Shuto, S. Kobayashi, Y. Yamaguchi, T. Nagamine, Y. Nakajima, K. Inagaki-Tanimura, and H. Sugihara, HLA analysis of immune checkpoint inhibitor-induced and idiopathic isolated ACTH deficiency*.* Pituitary, 2022. 25(4): p. 615-621. <https://doi.org/10.1007/s11102-022-01231-1>.

36. Oristrell, G., J. Baneras, J. Ros, and E. Munoz, Cardiac tamponade and adrenal insufficiency due to pembrolizumab: a case report*.* Eur Heart J Case Rep, 2018. 2(2): p. yty038. <https://doi.org/10.1093/ehjcr/yty038>.

37. Percik, R., G. Shlomai, A. Tirosh, A. Tirosh, R. Leibowitz-Amit, Y. Eshet, G. Greenberg, A. Merlinsky, E. Barhod, Y. Steinberg-Silman, and T. Sella, Isolated autoimmune adrenocorticotropic hormone deficiency: From a rare disease to the dominant cause of adrenal insufficiency related to check point inhibitors*.* Autoimmun Rev, 2020. 19(2): p. 102454. <https://doi.org/10.1016/j.autrev.2019.102454>.

38. Pierrard, J., B. Petit, S. Lejeune, and E. Seront, Isolated adrenocorticotropic hormone (ACTH) deficiency and Guillain-Barre syndrome occurring in a patient treated with nivolumab*.* BMJ Case Rep, 2019. 12(8). <https://doi.org/10.1136/bcr-2019-230848>.

39. Porntharukchareon, T., B. Tontivuthikul, N. Sintawichai, and P. Srichomkwun, Pembrolizumab- and ipilimumab-induced diabetic ketoacidosis and isolated adrenocorticotropic hormone deficiency: a case report*.* J Med Case Rep, 2020. 14(1): p. 171. <https://doi.org/10.1186/s13256-020-02502-w>.

40. Sakaguchi, C., S. Yano, K. Ashida, N. Wada, K. Ohe, H. Nagata, Y. Matsuda, S. Sakamoto, R. Sakamoto, K. Ohnaka, H. Uchi, M. Furue, M. Nomura, and Y. Ogawa, A Case of Acute Exacerbation of Chronic Adrenal Insufficiency Due to Ipilimumab Treatment for Advanced Melanoma*.* Am J Case Rep, 2019. 20: p. 106-110. <https://doi.org/10.12659/AJCR.913021>.

41. Sato, Y., Y. Tanaka, M. Hino, M. Seike, and A. Gemma, A case of nivolumab-induced isolated adrenocorticotropic hormone (ACTH) deficiency*.* Respir Med Case Rep, 2019. 26: p. 223-226. <https://doi.org/10.1016/j.rmcr.2019.01.021>.

42. Seki, T., A. Yasuda, M. Oki, N. Kitajima, A. Takagi, N. Nakajima, A. Miyajima, and M. Fukagawa, Secondary Adrenal Insufficiency Following Nivolumab Therapy in a Patient with Metastatic Renal Cell Carcinoma*.* Tokai J Exp Clin Med, 2017. 42(3): p. 115-120

43. Shrotriya, S., M.P. Rai, A. Alratroot, and E. Sarzynski, Delayed Presentation of Isolated Adrenocorticotropin Insufficiency after Nivolumab Therapy for Advanced Non-small-cell lung carcinoma (NSCLC)*.* BMJ Case Rep, 2018. 2018. <https://doi.org/10.1136/bcr-2018-225048>.

44. Suzuki, K., T. Terakawa, J. Furukawa, K. Harada, N. Hinata, Y. Nakano, and M. Fujisawa, Nivolumab-induced Adrenal Insufficiency in Patients With Renal Cell Carcinoma*.* J Immunother, 2020. 43(1): p. 38-42. <https://doi.org/10.1097/CJI.0000000000000299>.

45. Takaya, K., M. Sonoda, A. Fuchigami, and T. Hiyoshi, Isolated Adrenocorticotropic Hormone Deficiency Caused by Nivolumab in a Patient with Metastatic Lung Cancer*.* Intern Med, 2017. 56(18): p. 2463-2469. <https://doi.org/10.2169/internalmedicine.8548-16>.

46. Takebayashi, K., A. Ujiie, M. Kubo, S. Furukawa, M. Yamauchi, H. Shinozaki, T. Suzuki, R. Naruse, K. Hara, T. Tsuchiya, and T. Inukai, Isolated Adrenocorticotropic Hormone Deficiency and Severe Hypercalcemia After Destructive Thyroiditis in a Patient on Nivolumab Therapy With a Malignant Melanoma*.* J Clin Med Res, 2018. 10(4): p. 358-362. <https://doi.org/10.14740/jocmr3257w>.

47. Takeno, A., M. Yamamoto, M. Morita, S. Tanaka, I. Kanazawa, M. Yamauchi, S. Kaneko, and T. Sugimoto, Late-onset isolated adrenocorticotropic hormone deficiency caused by nivolumab: a case report*.* BMC Endocr Disord, 2019. 19(1): p. 25. <https://doi.org/10.1186/s12902-019-0335-x>.

48. Tanabe, J., N. Watanabe, M. Ito, K. Kanasaki, and K. Tanabe, Differentiating Immune-Related Adrenal Insufficiency From Low Cardiac Output Syndrome: A Case Report*.* Cureus, 2022. 14(11): p. e31349. <https://doi.org/10.7759/cureus.31349>.

49. Tanaka, S., M. Kushimoto, T. Nishizawa, M. Takubo, K. Mitsuke, J. Ikeda, M. Fujishiro, K. Ogawa, I. Tsujino, Y. Suzuki, and M. Abe, Isolated ACTH deficiency during single-agent pembrolizumab for squamous cell lung carcinoma: a case report*.* Clin Diabetes Endocrinol, 2020. 6: p. 1. <https://doi.org/10.1186/s40842-019-0092-9>.

50. Yamagata, S., K. Kageyama, S. Takayasu, Y. Asari, K. Makita, K. Terui, and M. Daimon, Progression of Hypopituitarism and Hypothyroidism after Treatment with Pembrolizumab in a Patient with Adrenal Metastasis from Non-small-cell Lung Cancer*.* Intern Med, 2019. 58(24): p. 3557-3562. <https://doi.org/10.2169/internalmedicine.3008-19>.

51. Yamauchi, I., D. Taura, T. Hakata, H. Fujita, K. Okamoto, Y. Ueda, T. Fujii, and N. Inagaki, Clinical features and thyroid dysfunction in adverse events involving the pituitary gland during PD-1 blockade therapy*.* Clin Endocrinol (Oxf), 2021. 94(2): p. 258-268. <https://doi.org/10.1111/cen.14349>.

52. Yano, S., K. Ashida, R. Sakamoto, C. Sakaguchi, M. Ogata, K. Maruyama, S. Sakamoto, M. Ikeda, K. Ohe, S. Akasu, S. Iwata, N. Wada, Y. Matsuda, Y. Nakanishi, M. Nomura, and Y. Ogawa, Human leucocyte antigen DR15, a possible predictive marker for immune checkpoint inhibitor-induced secondary adrenal insufficiency*.* Eur J Cancer, 2020. 130: p. 198-203. <https://doi.org/10.1016/j.ejca.2020.02.049>.

53. Zeng, M.F., L.L. Chen, H.Y. Ye, W. Gong, L.N. Zhou, Y.M. Li, and X.L. Zhao, Primary hypothyroidism and isolated ACTH deficiency induced by nivolumab therapy: Case report and review*.* Medicine (Baltimore), 2017. 96(44): p. e8426. <https://doi.org/10.1097/MD.0000000000008426>.

54. Zhu, Y., H.H. Wu, and W. Wang, A case of small-cell lung cancer with adrenocorticotropic hormone deficiency induced by nivolumab*.* Onco Targets Ther, 2019. 12: p. 2181-2186. <https://doi.org/10.2147/OTT.S194094>.

55. Rai, M. and M. Go, Nivolumab Induced Adrenal Insufficiency: Rare Side-effect of a New Anti-cancer Therapy - Immune-checkpoint Inhibitors*.* Cureus, 2020. 12(4): p. e7625. <https://doi.org/10.7759/cureus.7625>.

56. Sekizaki, T., H. Kameda, C. Oba, K. Yong Cho, A. Nakamura, H. Miyoshi, T. Osawa, N. Shinohara, and T. Atsumi, Nivolumab-induced hypophysitis causing secondary adrenal insufficiency after transient ACTH elevation*.* Endocr J, 2019. 66(10): p. 937-941. <https://doi.org/10.1507/endocrj.EJ19-0076>.

57. Thapi, S., A. Leiter, M. Galsky, and E.J. Gallagher, Recovery from secondary adrenal insufficiency in a patient with immune checkpoint inhibitor therapy induced hypophysitis*.* J Immunother Cancer, 2019. 7(1): p. 248. <https://doi.org/10.1186/s40425-019-0729-3>.

58. Kastrisiou, M., F.L. Kostadima, A. Kefas, G. Zarkavelis, N. Kapodistrias, E. Ntouvelis, D. Petrakis, A. Papadaki, A. Vassou, and G. Pentheroudakis, Nivolumab-induced hypothyroidism and selective pituitary insufficiency in a patient with lung adenocarcinoma: a case report and review of the literature*.* ESMO Open, 2017. 2(4): p. e000217. <https://doi.org/10.1136/esmoopen-2017-000217>.

59. Chang, J., J. Tran, D. Kamel, and A. Basu, Nivolumab-induced hypophysitis leading to hypopituitarism and secondary empty sella syndrome in a patient with non-small cell lung cancer*.* BMJ Case Rep, 2019. 12(3). <https://doi.org/10.1136/bcr-2018-228135>.

60. Newman, C., O. Kgosidalwa, O.A. Hakami, C. Kennedy, L. Grogan, and A. Agha, Multiple endocrinopathies, hypercalcaemia and pancreatitis following combined immune checkpoint inhibitor use- case report and review of literature*.* BMC Endocr Disord, 2021. 21(1): p. 33. <https://doi.org/10.1186/s12902-021-00693-x>.

61. Iglesias, P., J.C. Sanchez, and J.J. Diez, Isolated ACTH deficiency induced by cancer immunotherapy: a systematic review*.* Pituitary, 2021. 24(4): p. 630-643. <https://doi.org/10.1007/s11102-021-01141-8>.

62. Kobayashi, T., S. Iwama, Y. Yasuda, N. Okada, T. Okuji, M. Ito, T. Onoue, M. Goto, M. Sugiyama, T. Tsunekawa, H. Takagi, D. Hagiwara, Y. Ito, H. Suga, R. Banno, K. Yokota, T. Hase, M. Morise, N. Hashimoto, M. Ando, Y. Fujimoto, H. Hibi, M. Sone, Y. Ando, M. Akiyama, Y. Hasegawa, and H. Arima, Pituitary dysfunction induced by immune checkpoint inhibitors is associated with better overall survival in both malignant melanoma and non-small cell lung carcinoma: a prospective study*.* J Immunother Cancer, 2020. 8(2). <https://doi.org/10.1136/jitc-2020-000779>.

63. Lin, S.H., A. Zhang, L.Z. Li, L.C. Zhao, L.X. Wu, and C.T. Fang, Isolated adrenocorticotropic hormone deficiency associated with sintilimab therapy in a patient with advanced lung adenocarcinoma: a case report and literature review*.* BMC Endocr Disord, 2022. 22(1): p. 239. <https://doi.org/10.1186/s12902-022-01151-y>.

64. Fujita, Y., H. Bando, G. Iguchi, K. Iida, H. Nishizawa, K. Kanie, K. Yoshida, R. Matsumoto, K. Suda, H. Fukuoka, W. Ogawa, and Y. Takahashi, Clinical Heterogeneity of Acquired Idiopathic Isolated Adrenocorticotropic Hormone Deficiency*.* Front Endocrinol (Lausanne), 2021. 12: p. 578802. <https://doi.org/10.3389/fendo.2021.578802>.

65. Min, L., A. Vaidya, and C. Becker, Association of ipilimumab therapy for advanced melanoma with secondary adrenal insufficiency: a case series*.* Endocr Pract, 2012. 18(3): p. 351-5. <https://doi.org/10.4158/EP11273.OR>.

66. Bai, X., X. Chen, X. Wu, Y. Huang, Y. Zhuang, Y. Chen, C. Feng, and X. Lin, Immune checkpoint inhibitor-associated pituitary adverse events: an observational, retrospective, disproportionality study*.* J Endocrinol Invest, 2020. 43(10): p. 1473-1483. <https://doi.org/10.1007/s40618-020-01226-4>.

67. Iglesias, P., I. Peiro, B. Biagetti, M. Paja-Fano, D.A. Cobo, C. Garcia Gomez, M. Mateu-Salat, I. Genua, M. Majem, M. Riudavets, J. Gavira, C. Lamas, A. Fernandez Pombo, F. Guerrero-Perez, C. Villabona, J.M. Cabezas Agricola, S.M. Webb, and J.J. Diez, Immunotherapy-induced isolated ACTH deficiency in cancer therapy*.* Endocr Relat Cancer, 2021. 28(12): p. 783-792. <https://doi.org/10.1530/ERC-21-0228>.

68. Cui, K., Z. Wang, Q. Zhang, and X. Zhang, Immune checkpoint inhibitors and adrenal insufficiency: a large-sample case series study*.* Ann Transl Med, 2022. 10(5): p. 251. <https://doi.org/10.21037/atm-21-7006>.

69. Hanna, R.M., U. Selamet, P. Bui, S.F. Sun, O. Shenouda, N. Nobakht, M. Barsoum, F. Arman, and A. Rastogi, Acute Kidney Injury after Pembrolizumab-Induced Adrenalitis and Adrenal Insufficiency*.* Case Rep Nephrol Dial, 2018. 8(2): p. 171-177. <https://doi.org/10.1159/000491631>.

70. Juszczak, A., A. Gupta, N. Karavitaki, M.R. Middleton, and A.B. Grossman, Ipilimumab: a novel immunomodulating therapy causing autoimmune hypophysitis: a case report and review*.* Eur J Endocrinol, 2012. 167(1): p. 1-5. <https://doi.org/10.1530/EJE-12-0167>.

71. Ida, H., Y. Goto, J. Sato, S. Kanda, Y. Shinno, R. Morita, S. Murakami, Y. Matsumoto, T. Yoshida, H. Horinouchi, Y. Fujiwara, N. Yamamoto, T. Fukuda, K. Ohashi, and Y. Ohe, Clinical characteristics of adrenal insufficiency as an immune-related adverse event in non-small-cell lung cancer*.* Med Oncol, 2020. 37(4): p. 30. <https://doi.org/10.1007/s12032-020-01357-x>.

72. Otsubo, K., K. Nakatomi, R. Furukawa, K. Ashida, Y. Yoneshima, Y. Nakanishi, and I. Okamoto, Two cases of late-onset secondary adrenal insufficiency after discontinuation of nivolumab*.* Ann Oncol, 2017. 28(12): p. 3106-3107. <https://doi.org/10.1093/annonc/mdx497>.

73. Gu, Y.C., Y. Liu, C. Xie, and B.S. Cao, [Pituitary immune-related adverse events induced by programmed cell death protein 1 inhibitors in advanced lung cancer patients: A report of 3 cases]*.* Beijing Da Xue Xue Bao Yi Xue Ban, 2022. 54(2): p. 369-375. <https://doi.org/10.19723/j.issn.1671-167X.2022.02.027>.

74. Boudjemaa, A., G. Rousseau-Bussac, and I. Monnet, Late-Onset Adrenal Insufficiency More Than 1 Year after Stopping Pembrolizumab*.* J Thorac Oncol, 2018. 13(3): p. e39-e40. <https://doi.org/10.1016/j.jtho.2017.10.023>.

75. Nagasaka, M., N. Abdallah, J. Samantray, and A. Sukari, Is this really just "fatigue"? A case series of immune-related central adrenal insufficiency secondary to immune checkpoint inhibitors*.* Clin Case Rep, 2018. 6(7): p. 1278-1281. <https://doi.org/10.1002/ccr3.1567>.
